# Clinical and epidemiological features of heart-hand syndrome, an updated analysis in China

**DOI:** 10.1186/s12891-020-03813-1

**Published:** 2020-11-25

**Authors:** Yaobin Yin, Jianguang Ji, Junhui Zhao, Shanlin Chen, Wen Tian

**Affiliations:** 1 Department of Hand Surgery, Beijing Ji Shui Tan Hospital, Xin jie kou dong jie 31, Xi Cheng Qu, Beijing, 100035 China; 2 Center for Primary Health Care Research, Lund University, Malmö, Sweden; 3 Clinical Research Centre (CRC), Skåne University Hospital, Building 28, floor 11, Jan Waldenströms gata 35, SE-205 02 Malmö, Sweden

**Keywords:** Heart-hand syndrome (HHS), Congenital malformations, Clinical epidemiology, Classification

## Abstract

**Background:**

The purpose of this study was to prospectively recruit patients treated with limb malformation and to explore the prevalence and the clinical and epidemiological features of Heart-Hand Syndrome (HHS) in China.

**Methods:**

The consecutive patients treated for congenital upper limb malformation in Beijing Ji Shui Tan Hospital from October 1st, 2016 to October 1st, 2019 were prospectively recruited. We reviewed the patients’ medical records and identified patients with abnormal electrocardiogram (ECG) and/or abnormal ultrasonic cardiogram as well as their basic demographic and clinical characteristics.

**Results:**

A total 1653 (1053 male and 600 female) patients with congenital upper extremity malformations were prospectively recruited. Among them, 200 (12.1%) had abnormal ultrasonic cardiogram (181patients, 10.9%) and/or abnormal ECG (19 patients, 1.1%). The commonest type of abnormal heart structure was atrial septal defect (69/181 38.1%), and the commonest abnormal ECG was wave patterns (7/19, 36.8%). HHS patients had a higher comorbidity rate (11%) than non-HHS patients (6.9%). Patients with HHS were classified into four groups by the types of congenital upper extremity malformations, among which the most common group was thumb type (121/200, 60.5%).

**Conclusions:**

HHS occurred frequently among patients with congenital upper extremity malformation in China, particularly for those with multiple congenital malformations. The commonest type of hand malformations of HHS patients was thumb malformation.

## Background

Heart-hand syndrome (HHS) is characterized by the co-occurrence of a limb malformation and a congenital cardiac disease, presenting with substantial clinical and genetic heterogeneity [1]. HHS is a broad category of diseases which might be included in many different syndromes, such as the vertebral abnormalities, anal atresia, cardiac abnormalities, tracheoesophageal fistula and/or esophageal atresia, renal agenesis and dysplasia, and limb defect syndrome (VACTERL syndrome) [2], the “long-thumb” brachydactyly syndrome [3], thrombocytopenia-absent radius syndrome(TRS) [4], the Fanconi anemia syndrome [5], et al. Most congenital upper extremity malformations require surgical treatment, it is thus necessary to have a full understanding of the potential complications, such as simultaneous congenital heart disease, during anesthesia and operation.

As the mandatory policy of pre-marital medical examination has stopped and the one-child policy has been ended in China recently, the number of patients with congenital upper extremity malformation has been increased as a consequence of the changed policies [6]. In our previous retrospective study, we found that the prevalence of HHS was around 11.8% among 1462 patients treated with congenital upper extremity malformations [7]. In light of the increased number of patients with congenital upper extremity malformation in China, we expected that more HHS cases might be diagnosed in Chinese patients. We thus planned this cohort study and aimed (1) to prospectively recruit patients with congenital upper extremity malformation and explore the prevalence of HHS, (2) to update the characteristics and the clinical features of HHS in China, (3) to make a new classification for HHS based on the clinical characteristics of hand malformations as previous classifications of HHS do not meet the need of HHS patients in China.

## Methods

The Ethics Committee at Beijing Ji Shui Tan Hospital approved this study. All methods were carried out in accordance with relevant regulations and guidelines, and written consent to participate was obtained from the parents/guardians of the children included in this study (children are considered anyone under the age of 16).

Beijing Ji Shui Tan Hospital is a national orthopedics center in China. All the patients treated for congenital upper extremity malformation in this hospital between October 1st, 2016 and October 1st, 2019 were prospectively recruited in this study. The medical records and the results of electrocardiogram (ECG) and ultrasonic cardiogram were reviewed separately by two clinicians. Patients with abnormal ECG and/or abnormal ultrasonic cardiogram were identified.

We further classified upper extremity malformation according to the Swanson upper extremity malformation classification [8], and analyzed the proportion of HHS for each type of upper extremity malformation. We also analyzed the different abnormal ultrasonic cardiogram and ECG for each type of upper extremity malformations. We explored the correlation of HHS with the blood type and comorbidity among patients with upper extremity malformations. We further made a new classification of HHS based on the type of hand malformations among Chinese patients.

### Statistics

Descriptive data were presented as number and percentage for the categorical variables. *P* value was examined using chi-square test for categorical variables. SAS version 9.2 (SAS Institute, Cary, NC, USA) was used for statistical analyses.

## Results

A total 1653 (1053 male and 600 female) patients with congenital upper extremity malformations were recruited and treated in the Hand Surgery Department of Beijing Ji Shui Tan Hospital between October 1st, 2016, and October 1st, 2019 (Table 1). Among them, duplication (type III Swanson upper extremity malformation classification) was the most common type. Blood samples were available for 884 patients. Except for congenital cardiac disease, 122 patients (7.4%) were diagnosed with other comorbidities and the lower extimity malformations were the most common.

**Table 1:** Basic demographic and clinical characteristics among patients treated with congenital upper limb malformation

Characteristic	Number	%
**Total**	1653	100.0
**Age**		
0–4	1328	80.3
5–9	202	12.3
10+	123	7.4
Median	2	
**Gender**		
Male	1053	63.7
Female	600	36.3
**Hand abnormality**		
I Failure of formation of parts (arrest of development)	70	4.2
II Failure of differentiation (separation) of parts	537	32.5
III Duplication	921	55.7
IV Overgrowth	10	0.6
V Undergrowth	79	4.8
VI Congenital constriction band syndrome	36	2.2
**Blood type**		
A	260	15.7
B	276	16.7
O	262	15.8
AB	86	5.2
Missing	769	46.5
**Comorbidity except for heart**	122	7.4
Toes malformation	77	4.7
Ankle malformation	16	1.0
Knee malformation	5	0.3
Hip malformation	4	0.2
Spine malformation	5	0.3
Facial malformation	10	0.6
Thoracic malformation	2	0.1
Kidney malformation	2	0.1
Anemia	1	0.1

There were 200 patients (200/1653) who met the clinical diagnosis criteria of HHS. One hundred eighty-one patients had abnormal heart structure and 19 patients only had abnormal ECG. In Table 2 we present the distribution of various congenital cardiac diseases among different types of upper limb malformations. There was a significant difference for congenital cardiac disease among the different types of upper limb malformations (*p* < 0.05). Type I Swanson upper extremity malformation classification had the highest prevalence of congenital cardiac diseases. The commonest type of abnormal heart structure was atrial septal defect (69/181, 38.1%), followed by tricuspid regurgitation (65/181, 36.9%). The commonest abnormal result of ECG was abnormal wave patterns (7/19, 36.8%).

**Table 2:** Prevalence of congenital heart defect stratified by different hand abnormality

Characteristic	I Failure of formation of parts (arrest of development	II Failure of differentiation (separation)	III Duplication	IV Overgrowth	V Undergrowth	VI Congenital constriction band syndrome	Total
No. patient	70	537	921	10	79	36	1653
No. congenital heart defect	18	60	103	0	13	6	200
%	25.7	11.2	11.2	0.0	16.5	16.7	12.1
Atrial septal defect	7	16	43	0	2	1	69
Ventricular septal defect	3	1	6	0	0	0	10
Multiple valve regurgitation	1	4	2	0	1	0	8
Patent ductus arteriosus	0	2	4	0	1	2	9
Mitral regurgitation	1	0	4	0	1	1	7
Tricuspid regurgitation	2	27	32	0	3	1	65
Aortic valve regurgitation	0	1	2	0	0	0	3
Persistent left superior vena cava	2	1	2	0	1	1	7
Tetralogy of Fallot	0	0	1	0	0	0	1
Pulmonary artery sling	0	0	1	0	0	0	1
Pulmonary stenosis	0	1	0	0	0	0	1
Conduction block	2	1	1	0	1	0	5
Abnormal wave patterns	0	4	1	0	2	0	7
Premature beat	0	2	2	0	1	0	5
Tachycardia.	0	0	2	0	0	0	2
*P* value	0.009						

In Table 3, we present the distribution of blood type among patients with congenital upper limb malformations and the prevalence of HHS. Patients with blood type B had the highest prevalence of HHS (13.4%), but there was no significant difference among different blood types.

**Table 3:** Prevalence of congenital heart defect stratified by different blood type

Blood type	Total	HHS	%	*P* value
A	260	33	12.7	0.94
B	276	37	13.4	
O	262	30	11.5	
AB	86	10	11.6	
Missing	769	90	11.7	
Total	1653	200	12.1	

In Table 4, we analyzed the distribution of different comorbidities among patients with congenital upper limb malformations stratified by HHS diagnosis. HHS patients had a higher rate of comorbidity (11%) than non-HHS patients (6.9%) (*P* < 0.05).

**Table 4:** Prevalence of HHS and non-HHS stratified by comorbidity

Comorbidity	HHS		Non-HHS		*P* value
	No.	%	No.	%	
Lower extremity malformation	15	7.5	87	6.0	
Spine malformation	2	1.0	3	0.2	
Facial malformation	3	1.5	7	0.5	
Thoracic malformation	0	0.0	2	0.1	
Kidney malformation	2	1.0	0	0.0	
Anemia	0	0.0	1	0.1	
Total	22	11.0	100	6.9	0.001

According to our patients, we made a new classification of HHS based on the malformations of the hand. The new classification included a total of 4 types: thumb type, finger type, contracture type, and rare type, as shown in Table 5. The commonest type was thumb type, which accounted for 60.5% of patients with HHS.

**Table 5:** HHS classification according to the hand malformations

Type	Subtype^a^		
	a	b	%
Type1 Thumb type (thumb hypoplasia, thumb duplication, triphalangeal thumb, et al)	113	8	60.5%
Type2 Finger type (syndactyly, brachydactyly, symbrachydactyly, et al)	45	8	26.5%
Type3 Contracture type (single or multiple congenital joint contractures)	22	3	12.5%
Type4 Rare type (could not classify into malformations mentioned above)	1	0	0.5%

^a^Subtype a: an abnormal heart structure was discovered by ultrasonic cardiogram, associated with normal EGC or abnormal ECG. Subtype b: only abnormal result of ECG was discovered

## Discussion

In this prospective cohort study, we identified a total of 1653 patients with congenital upper extremity malformations.We found that the prevalence of HHS was 12.1%, which showed a similar result as in our previous retrospective study [7]. Type I congenital upper limb malformations (Failure of formation of parts) was most likely to present with congenital cardiac disease, which was also in line with the result of our previous study. In this study, 60.5% of HHS patients had thumb malformations, similar to what we found in the previous study (69.2%). Some studies have shown that the thumb is remarkably different from the other four digits and developed in a different molecular environment, which provides the underlying mechanisms for the strong association between thumb malformations and HHS [9, 10], suggesting that genes related to thumb formation might be related to HHS.

Our previous study was based on patients treated with upper extremity malformations from October 1st, 2013 to October 1st, 2016 in the same hospital. The number of beds and surgeons in this hospital did not change after the previous study. However, the number of patients who were treated with upper extremity was increased by 13% ((1653–1462)/1462 = 0.13). The underlying reasons are not clear as we did not find a change of the referral pattern of patients and and no therapeutic centers nearby were closed. This phenomenon may be related to the Chinese policy changed from “One family, one child” to “one family, two children”. The increased rate of upper extremity malformations might be due to the increased number of elderly pregnant women associated with this policy change [11].

HHS is well documented since first reported by Kato in 1924 [12]. It has been reported by many others subsequently [13, 14]. HHS includes the most common type – HHS I: Holt–Oram syndrome [13] (congenital heart disease and radial ray anomalies) and several rare types – HHS II: Tabatznik syndrome (arrhythmias and brachytelephalangy) [15], HHS III: Spanish type (arrhythmia and brachydactyly type C) [16], and a potential HHS IV: Slovenian type (arrhythmia, dilated cardiomyopathy, and brachydactyly) [17]. However, there were only 16 patients (8%) in this study who could be classified according to previous classifications. These 16 patients met the clinical diagnosis of Holt–Oram syndrome. ASD (7 patients, 44%) was the commonest cardiovascular anomaly for these patients, the rate of which was lower than that of the study reported by Wall, L.B. (ASD 53%) [18].

Most HHS patients in this study could not be classified according to current knowledge, suggesting that there might be ethnic variance of HHS. It is thus necessary to create a new classification for HHS in China. According to the clinical characteristics of these patients, we made a new classification. This new classification was based on the malformations of the hand and could be used easily. The commonest type of hand malformations of HHS patients in this study was related to thumb malformation (121, 60.5%), including thumb hypoplasia, thumb duplication, triphalangeal thumb, et al. Lam et al. found that preaxial polydactyly (thumb duplication) and forearm radial deficiencies may share a common developmental origin [19]. So, we put all malformations related to thumb into one type. The second type (53, 26.5%) is related to malformations including syndactyly, brachydactyly, and symbrachydactyly. This type included previous HHS II, HHS III, and HHS IV. The third type was related to congenital contractures (25 12.5%). Joint movement limitations in HHS might be considered close to the classical joint contractures observed in Emery–Dreifuss muscular dystrophy, which was LMNA generelated disease [20]. Renou et al. reported an intronic LMNA mutation that co-segregated with the HHS clinical features [17]. The fourth type included other hand malformations except for the malformations mentioned above. This new classification based on clinical characteristics is easy to use and could cover all of the HHS patients in this study. However, we cannot identify the molecular causes regarding different subcategories based on current evidence and further studies are higher needed.

We analyzed the distribution of blood types of HHS patients. Blood type B had the highest morbidity rate (13.4%), but there was no significant difference between blood types. We need further research on this field because around half of the patients did not attend blood type test. Further studies with enough samples are needed to explore the association of HHS with blood type in detail. The gene related to ABO type has been identified and resides on chromosome 9 at the band 9q34. 2 with 7 exons, it is thus higher possible to identify the underlying genes of HHS based on genetic linkage analyses if we can observe the association between specific blood type with HHS. In this study, 122 patients (7.4%) had comorbidity conditions except for the heart. The lower extremity malformations were the commonest. HHS patients had a much higher morbidity rate (11%) than non-HHS patients (6.9%) (*P* < 0.05). The comorbidity conditions were reported previously [21, 22], but this is the first time to report the morbidity rate for HHS. It is highly necessary to inform patients who had multiple congenital malformations because the prevalence of HHS was higher than that with only upper extremity malformations.

There were a few limitations in this study. First, all the patients were recruited from the hand surgery department in one Chinese national orthopedics center, which might not be a comprehensive representative of the Chinese population. In addition, the classification that we proposed in this study needs to be confirmed by other studies.

## Conclusions

In summary, the prevalence of HHS among patients with congenital upper extremity malformations is relatively high in China, especially for patients with multiple congenital malformations. The commonest type of hand malformations of HHS patients was related to thumb malformation.

## Data Availability

The datasets used and/or analysed during the current study are available from the corresponding author on reasonable request.
